# Cross-feeding interactions between *Fusobacterium nucleatum* and the glycan forager *Segatella oris*

**DOI:** 10.1128/msystems.00922-25

**Published:** 2026-01-21

**Authors:** Joshua R. Fletcher, Areej Malik, Jacob Driggers, Ryan C. Hunter

**Affiliations:** 1 Department of Population Health and Pathobiology, North Carolina State University College of Veterinary Medicine 70727, Raleigh, North Carolina, USA; 2 Department of Microbiology & Immunology, Jacobs School of Medicine and Biomedical Sciences, University at Buffalo 12292 , Buffalo, New York, USA; University of California San Diego, La Jolla, California, USA

**Keywords:** *Fusobacterium nucleatum*, *Segatella oris*, *Prevotella oris*, cross-feeding, glycans, microbiome, chronic airway disease, transcriptomics

## Abstract

*Fusobacterium nucleatum* is a common member of the oral microbiota frequently associated with extra-oral infections and diverse polymicrobial environments, including chronic airway diseases and colorectal tumors. Yet, its interactions with co-colonizing microbiota remain poorly defined. Here, we investigate cross-feeding interspecies dynamics between *F. nucleatum* and *Segatella oris,* a glycan-foraging anaerobe enriched in the airways, oral cavity, and gastrointestinal tumors. Using broth cultures, cell-free supernatants, and co-culture on primary human airway epithelial cells, we identify microbe-microbe and microbe-host interactions that shape nutrient acquisition, biofilm formation, gene expression, and host responses. While mucin or *S. oris* supernatants modestly enhanced *F. nucleatum* growth, both conditions triggered transcriptional remodeling, including induction of the *nan* operon for sialic acid catabolism, suggesting reliance on glycan degradation by *S. oris*. Conversely, *S. oris* exhibited differential expression of multiple polysaccharide utilization loci (PULs) when exposed to *F. nucleatum* or its metabolites. Biofilm formation by *F. nucleatum* was strongly inhibited by *S. oris* and its supernatants*,* indicative of metabolic regulation. Dual and triple RNA-seq revealed that epithelial responses were predominately shaped by *F. nucleatum,* with enrichment of inflammatory and cancer-associated pathways; however, co-colonization with *S. oris* modulated the expression of genes linked to the unfolded protein response and apoptosis, among others. These findings demonstrate that glycan-mediated cross-feeding and microbial interactions shape the physiology and pathogenic potential of *F. nucleatum* in mucosal environments. This work underscores the importance of modeling polymicrobial communities under host-relevant conditions to better understand pathobiont behavior at the epithelial interface.

**IMPORTANCE:**

*Fusobacterium nucleatum* is increasingly recognized as a pathobiont in mucosal diseases, including colorectal cancers and chronic airway infections, yet its functional interactions with co-colonizing microbiota remain poorly understood. Here, we demonstrate that *F. nucleatum* engages in bidirectional interactions with *Segatella oris,* a glycan-foraging anaerobe also enriched in mucin-rich environments. Through nutrient cross-feeding and transcriptional modulation, these interactions shape bacterial behavior and the host epithelial response. Notably, glycan degradation by *S. oris* enables *F. nucleatum* access to sialic acids, while *F. nucleatum* suppresses the expression of multiple polysaccharide utilization loci in *S. oris,* revealing a reciprocal ecological influence. Co-colonization of the airway epithelial surface also modulates gene expression linked to inflammation and cancer. These findings advance our understanding of polymicrobial dynamics at mucosal interfaces and highlight the importance of incorporating microbe-microbe-host interactions into reductionist models of infection and disease.

## INTRODUCTION

*Fusobacterium nucleatum* is a Gram-negative member of the oral microbiota that contributes to biofilm assembly, architecture, and community organization (1, 2). Although best known for its roles in dental plaque and periodontal disease, *F. nucleatum* is implicated in a range of extraoral infections and is frequently detected in tumors across multiple cancer types (3–6). While it can function as both a commensal and pathogen, *F. nucleatum* is often found in diverse polymicrobial host environments, including colorectal tumors and the airways of individuals with chronic rhinosinusitis (CRS), cystic fibrosis (CF), or chronic obstructive pulmonary disease (COPD) (7–14). This tendency raises questions about whether and how *F. nucleatum* interacts with co-colonizing microbiota and how these interactions shape its behavior and pathogenic potential. Equally important is understanding how *F. nucleatum* influences the activity of other taxa in these communities, many of which also exhibit context-dependent commensal or pathogenic behavior (15).

*Segatella oris* (formerly *Prevotella oris*) is also a member of the oral microbiota though less is known about its physiology or microbial interactions. Notably, *S. oris* supernatants inhibit the growth of the airway pathogen *Moraxella catarrhalis in vitro*, suggesting a connection between *S. oris* residence in the upper airways and colonization resistance against some pathogens (16). However, *S. oris* also exhibits pathogenic traits, including hemolysin production with activity against human erythrocytes (17). Like *F. nucleatum*, it is detected in extraoral infections, gastric tumors, CRS and CF airway mucus and is co-enriched with *F. nucleatum* in the oral microbiomes of cancer patients with chemotherapy-induced oral mucositis (9, 18, 19). More broadly, both *Fusobacterium* and *Prevotella* genera are co-detected in esophageal tumors (20), suggesting that cross-feeding interactions between these taxa may facilitate colonization and promote disease.

Although environmental conditions vary across infection sites, shared features such as host glycans, proteoglycans, and glycosaminoglycans are common. These features are particularly prevalent in mucus-laden airways of patients with chronic respiratory diseases and the mucus layer lining the intestinal tract, where *F. nucleatum* is frequently detected (21). *F. nucleatum* primarily ferments amino acids to generate ATP although glucose and fructose metabolism have also been reported (22–26). While it produces intracellular polyglucose granules *in vitro*, the genetic basis and functional significance of this pathway, including possible links to virulence, remain unclear (27). Notably, *F. nucleatum* lacks sialidases and polysaccharide utilization loci (PULs) but can scavenge sialic acids liberated by glycan foraging microbes (28), potentially enhancing community persistence through cross-feeding. In contrast, the genome of the *S. oris* type strain NCTC 13071 encodes 27 predicted PULs, likely involved in sensing, importing, and degrading of complex polysaccharides such as mucins and other host glycans (29, 30).

Given their frequent co-occurrence in mucin-rich environments *in vivo* and the possible complementarity of their metabolic repertoires, we hypothesized that *F. nucleatum* and *S. oris* engage in cross-feeding interactions. To test this, we examined how each species adjusts its growth and gene expression to mucin glycoproteins and each other’s metabolic byproducts. We found that while neither mucins nor *S. oris* supernatant substantially altered the growth of *F. nucleatum*, both conditions triggered significant transcriptional changes. Conversely, *S. oris* exhibited distinct growth dynamics and widespread transcriptional responses in mucin and *F. nucleatum* supernatants. Although *F. nucleatum* forms robust biofilms, biofilm formation was greatly reduced in *S. oris* supernatants and in co-culture despite biofilm growth in mucin or control media. We further extended these analyses using co-culture of each species, individually and together, on the apical surface of primary human airway epithelial cells to model aspiration-based colonization relevant to CF, CRS, and COPD. Dual (host-*S. oris,* host-*F. nucleatum*) and triple (host-*S*. o*ris-F. nucleatum*) RNA sequencing revealed that host-associated growth conditions strongly influence bacterial gene expression, including unexpected variability in *S. oris* PUL expression mediated by *F. nucleatum*. Host transcriptomic responses were primarily driven by *F. nucleatum,* with prominent induction of pro-inflammatory mediators *TNFA* and *TNFAIP2*, as well as several genes linked to cancer. Together, these data capture key information about bacterial interactions on relevant host cell types and highlight how co-colonizing, cross-feeding partners may modify each other’s physiology and influence host responses.

## MATERIALS AND METHODS

### Bacterial strains and media conditions

*Fusobacterium nucleatum* subsp. *nucleatum* strain ATCC 25586 and *Segatella oris* strain NCTC13071 were propagated anaerobically in a Coy anaerobic chamber (90% nitrogen, 5% carbon dioxide, 5% hydrogen) using BBL *Brucella* broth (BD) or 1.5% agar plates supplemented with hemin (250 μg/mL) and vitamin K (50 μg/mL) (Hardy Diagnostics). A semi-defined control medium was prepared by combining *Brucella* broth with minimal salts (50:50) as previously described (31). A minimal mucin medium (MMM) was generated by autoclaving porcine gastric mucin (PGM; Sigma) at 30 g/L in water, diluting to 15 g/L in 2× minimal salts, and combining 1:1 with *Brucella* broth to yield the final mucin-containing experimental medium (hereafter referred to as mucin medium), adapted from Flynn et al. (32) with dialysis steps omitted. Cell-free supernatants (CFS) were prepared in biological triplicate by culturing each strain for 48 h in mucin medium (10 mL) under anaerobic conditions. Cultures were centrifuged at 4,000 rpm at 4°C for 20 min, and the resulting supernatants were then passed through 0.22 μm filters. CFS were stored at −80°C and thawed once immediately prior to use.

### Growth curves

*F. nucleatum* and *S. oris* were grown overnight in *Brucella* broth supplemented with hemin (0.35 mg/mL) and vitamin K (0.05 mg/mL) and then subcultured 1:5 in fresh medium and grown for an additional 4 h. Optical density at 600 nm (OD600) was determined via spectrophotometry and adjusted to 0.01 in the respective growth medium (control, mucin, or cell-free mucin medium supernatants). Two hundred microliters of each culture was added to individual wells in a clear, flat-bottomed 96-well plate in technical triplicate across three biological replicates. Plates were sealed with a Breathe-Easy gas-permeable membrane and incubated in a Tecan Sunrise plate reader in the anaerobic chamber at 37°C for 72 h. OD600 was recorded hourly following 5 s of linear shaking. Viable bacteria were enumerated from 2 mL cultures at 0, 4, 8, and 24 h after inoculation by serial 10-fold dilution and plating onto *Brucella* agar supplemented with hemin and vitamin K. Plates were incubated anaerobically for 48 h prior to colony counting.

### Biofilm assays

Biofilm assays were performed in parallel using identical media conditions as described for growth curves, following the protocol of Merritt et al. (33). Briefly, microtiter plates were inoculated with each species individually or in co-culture and incubated anaerobically at 37°C for 48 h. Following incubation, plates were removed from the chamber and planktonic cultures were discarded. Plates were washed three times with 250 μL water, stained with 200 μL 0.1% crystal violet (in water) for 15 min, and then washed again three times and air-dried overnight. Crystal violet was solubilized with 200 μL of 30% acetic acid, and absorbance at 550 nm was determined using a BioTek Synergy H1 microplate reader. Wells containing sterile media were used as negative controls for background subtraction.

### Colonization of primary human airway epithelial monolayers

Normal human bronchial epithelial (NHBE) cells (Lonza Bioscience) obtained from healthy donors were expanded B-ALI Growth Basal Medium (Lonza) and seeded on 6.5 mm Transwell inserts (0.4 µm pore; STEMCELL Technologies). These cells produce mucus *in vitro*, primarily MUC5AC and MUC5B, making them excellent models for bacterial glycan foraging and cross-feeding (34, 35). Upon reaching confluency after ~2–4 days post-seeding, apical medium was removed, and basolateral medium was replaced with B-ALI Differentiation Basal Medium (Lonza). Cells were maintained at air-liquid interface (ALI) for 3–4 weeks at 37°C and 5% CO_2_ in a humidified incubator.

Overnight bacterial cultures were grown in *Brucella* broth supplemented with hemin and vitamin K. These were subcultured 1:5 in fresh medium and grown to OD600 = 0.5. Cultures were diluted 1:10 into infection medium (DMEM supplemented with 2% FBS, 10 mM HEPES, 0.1 mM nonessential amino acids, 4 mM L-glutamate, and 1 mM sodium pyruvate). One hundred microliters of bacterial suspension was applied to the apical surface of each Transwell and incubated for 4 h at 37°C under anaerobic conditions, resulting in an approximate MOI of 10:1 bacteria per host cell. After incubation, apical supernatants were gently removed by pipette, and co-cultures were maintained for an additional 20h prior to RNA extraction.

### RNA extraction

Each species was grown in 10 mL of control, mucin, and CFS media in 15 mL conical Falcon tubes for 24 h and collected by centrifugation at 4,000 rpm for 20 min at 4°C. Pellets were dissolved by gentle pipetting in 1 mL of TRIzol Reagent (ThermoFisher). For airway epithelial co-cultures, 250 μL TRIzol was added to the apical surface and a 1 mL pipette tip was used to scrape cellular material from the transwell surface. Scraping was performed four times per insert to yield 1 mL of total lysate in TRIzol. All TRIzol samples were incubated for 5 min at room temperature, followed by the addition of 200 μL chloroform, hand agitation for 15 s, followed by another 5-min incubation on the benchtop. Phase separation was performed by centrifugation at 12,000 rpm for 15 min at 4°C. The aqueous phase was mixed 1:1 with 95% ethanol, and RNA was column purified with the Zymo Clean & Concentrator-5 kit including an on-column DNase-I treatment according to manufacturer’s instructions.

### RNA sequencing and analysis

Total RNA from broth cultures was submitted to Seq Center (Pittsburgh, PA), where rRNA depletion was performed with the Illumina Stranded Total RNA Prep with Ribo-Zero Plus Microbiome kit prior to library preparation and sequencing (2 × 150 bp). RNA from bacterial and epithelial co-cultures was sent to SeqCoast Genomics (Portsmouth, NH). rRNA depletion was also performed on these samples prior to Illumina sequencing (2 × 150 bp). Sequencing was performed on the Illumina NextSeq2000 platform with a 300 cycle flow cell kit. Read quality was assessed using FastQC (https://www.bioinformatics.babraham.ac.uk/projects/fastqc/). Given the consistently high quality and the risk of biasing downstream analyses (36), no read trimming was performed.

For bacterial RNA seq, coding sequences for all annotated genes and riboswitches, gene names, and locus tags were extracted from the *F. nucleatum* ATCC 25586 (NZ_CP028101) and *S. oris* NCTC 17031 (NZ_LR134384) genomes in Geneious Prime (2024.0.7) and converted to FASTA format via the “Tabular-to-fasta” tool via the Galaxy server (https://usegalaxy.org/), and indexed with Salmon (37) for quasi-mapping of reads. For host RNA-seq, the *Homo sapiens* transcriptome (GRCh38.p14, release 45) was retrieved from Gencode (https://www.gencodegenes.org/human/release_45.html). Transcriptome indices were built in Salmon, to which reads were quasi-mapped. The quant.sf files generated by Salmon were imported into RStudio via the tximport package (38), and differential expression analysis was performed with DESeq2 (39). The threshold for differential expression was a log2 fold change ≥1 at an adjusted *P*-value <0.05. Code for each analysis is available at https://github.com/Hunter-Lab-UMN/Fletcher_FnSo_2025.

## RESULTS

### Growth and biofilm formation of *F. nucleatum* and *S. oris* are influenced by mucin and cross-feeding *in vitro*

**Fig 1:**
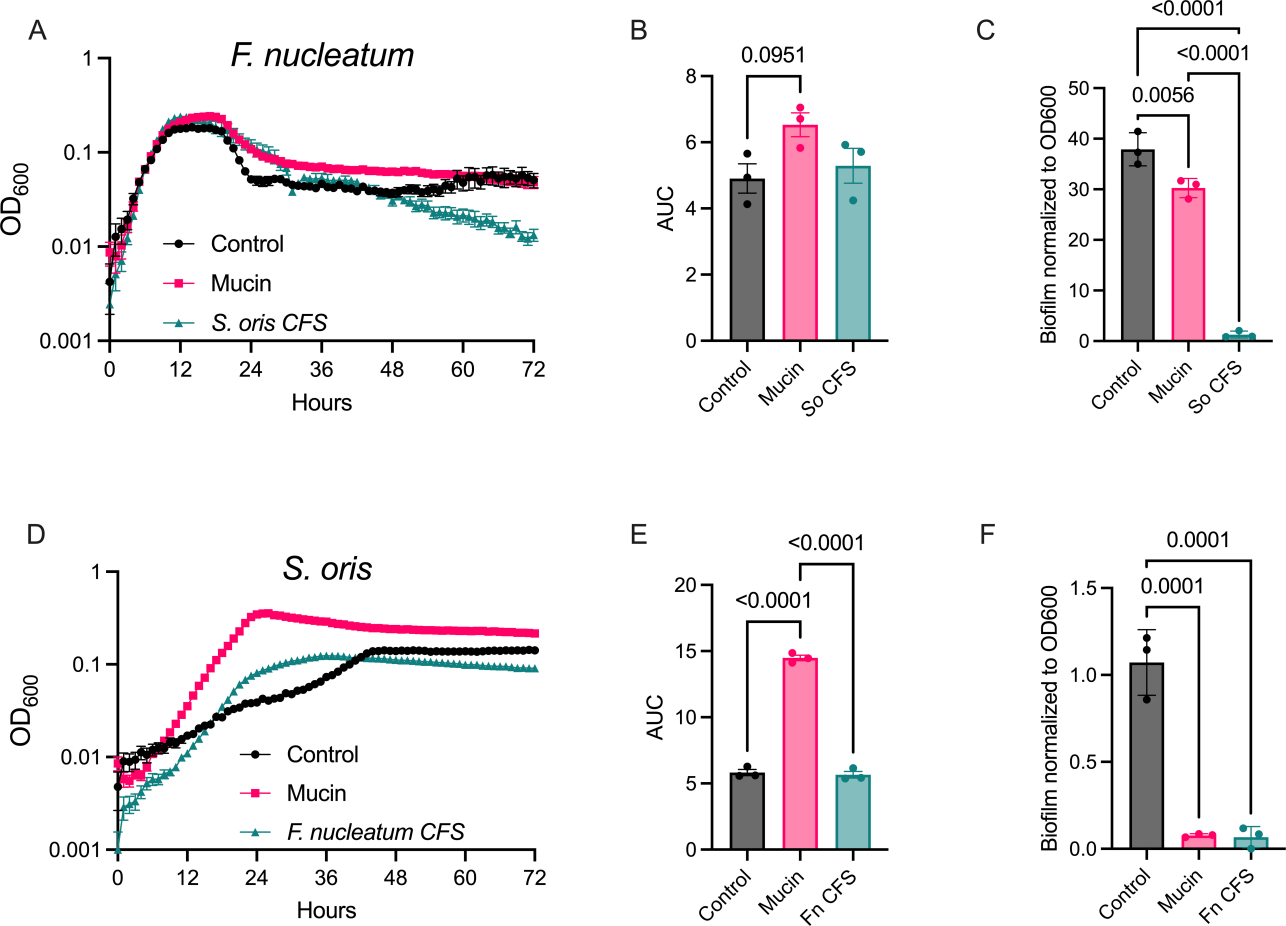
Mucin and interspecies metabolite exchange influence growth and biofilm formation of *F. nucleatum* and *S. oris*. (**A**) Growth curves of *F. nucleatum* in control medium, mucin medium, and *S. oris* cell-free supernatants (CFS) (*n* = 3). (**B**) Area under the curve (AUC) analysis for *F. nucleatum* growth. (**C**) Biofilm formation by *F. nucleatum* in each condition after 48 h, quantified by crystal violet staining. (**D**) Growth curves of *S. oris* in control medium, mucin medium, and *F. nucleatum* CFS. (**E**) AUC analysis for *S. oris* growth. (**F**) Biofilm formation by *S. oris* across all conditions. Data represent the mean of three biological replicates ± standard error of the mean (*n* = 3). Statistical significance in panels **B**, **C**, **E**, and **F** was determined using ordinary one-way ANOVA with Tukey’s multiple comparisons test. Error bars in panels **A** and **D** are the standard error of the mean (SEM).

*F. nucleatum* and *S. oris* were cultured under three conditions: (i) control medium, (ii) control medium supplemented with porcine gastric mucin (hereafter “mucin medium”), or (iii) cell-free supernatants (CFS) derived from each species grown in mucin medium for 48 h. In the control medium, *F. nucleatum* exhibited distinct growth phases with a pronounced exponential phase followed by stationary and death phases, with optical density remaining consistent after ~36 h. Exponential growth rates were similar across conditions; however, cultures grown in mucin medium or *S. oris* CFS reached higher stationary-phase densities and exhibited a slower decline during death phase compared to controls (~12–36 h; Fig. 1A). This elevated density in mucin medium was sustained until ~60 h, after which it declined to control levels. Area-under-curve (AUC) analysis reflected this trend, with mucin medium showing a modest, though not statistically significant, increase relative to the control (*P* = 0.0951; Fig. 1B). In contrast, *F. nucleatum* density in *S. oris* CFS declined sharply after 48 h, yielding an AUC comparable to the control. Experiments were repeated in larger culture volumes, and CFUs were enumerated at 0, 4, 8, and 24 h (Fig. S1A and B). Each strain grew well in all media, with *S. oris* CFUs being more consistent between conditions than was apparent during growth in microtiter plates.

Biofilm formation by *F. nucleatum* was robust in both control and mucin media, with a trend toward increased biofilm in mucin medium. However, biofilm growth was nearly abolished in *S. oris* CFS (Fig. 1C). Although nutrient depletion in *S. oris* CFS could partially contribute to reduced biofilm formation, this is unlikely given that *F. nucleatum* exhibited comparable planktonic growth in CFS and control medium. These findings suggest that *S. oris* produces one or more metabolites that inhibit *F. nucleatum* biofilm development. This suppression is unexpected given *F. nucleatum’s* well-established role as a so called bridging species in the highly structured and spatially stratified polymicrobial biofilms of the oral cavity (40).

In contrast, *S. oris* exhibited markedly different growth dynamics across conditions. Growth in control medium was slow with apparent diauxie following a brief stationary phase at ~24 h, a second exponential phase, and final plateau at ~40 h (Fig. 1D). This pattern may reflect initial depletion of readily metabolizable sugars followed by utilization of more complex structures such as mucin glycans, consistent with its predicted PUL repertoire. In mucin medium, *S. oris* grew much more rapidly, reaching a higher maximum density and exhibiting a threefold increase in AUC compared to the control (Fig. 1E). Likewise, diminished growth in *F. nucleatum* CFS could result from reduced availability of simple carbohydrates though the enhanced growth in mucin medium suggests that *S. oris* efficiently forages host glycans when preferred carbon sources are limited. These findings are consistent with genomic predictions of glycan utilization via PULs. Growth in *F. nucleatum* CFS yielded a faster growth rate than control medium but lower stationary phase density than mucin medium; AUC for CFS and control were comparable. Biofilm formation by *S. oris* was minimal in the control medium and nearly undetectable in mucin medium or CFS conditions (Fig. 1F). In dual-species cultures grown in control or mucin media, biofilms phenocopied the minimal single-species biofilms of *S. oris* (Fig. S1).

### *F. nucleatum* and *S. oris* transcriptomes are modulated by mucin medium, cross-feeding, and interactions with human airway epithelia

**Fig 2:**
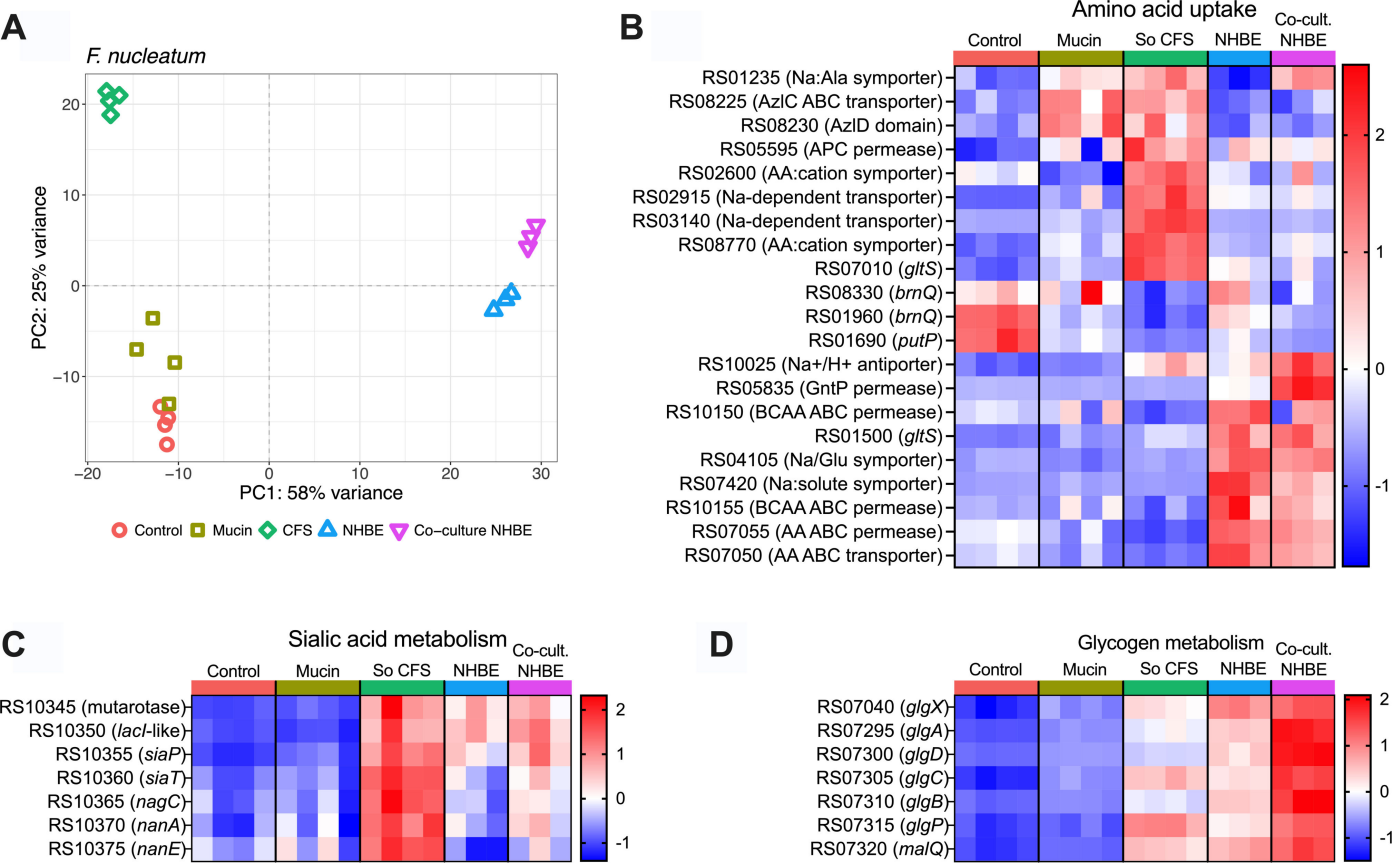
Transcriptomic responses of *F. nucleatum* to mucin, *S. oris* supernatants, and epithelial co-culture is (**A**) Principal component analysis (PCA) of *F. nucleatum* transcriptomes across control medium, mucin medium, CFS from *S. oris* grown in mucin medium, on normal human bronchial epithelia individually or in co-culture with *S. oris*. (**B**) Heatmap of amino acid transporter gene expression across conditions. (**C**) Expression of sialic acid catabolism (*nan* operon) genes. (**D**) Expression of glycogen metabolism genes (see Supplemental Files for complete gene lists). Each column in the heatmaps is an individual biological replicate, and each row is a transcript. Normalized read counts from DESeq2 are scaled by row and presented as Z-scores.

**Fig 3:**
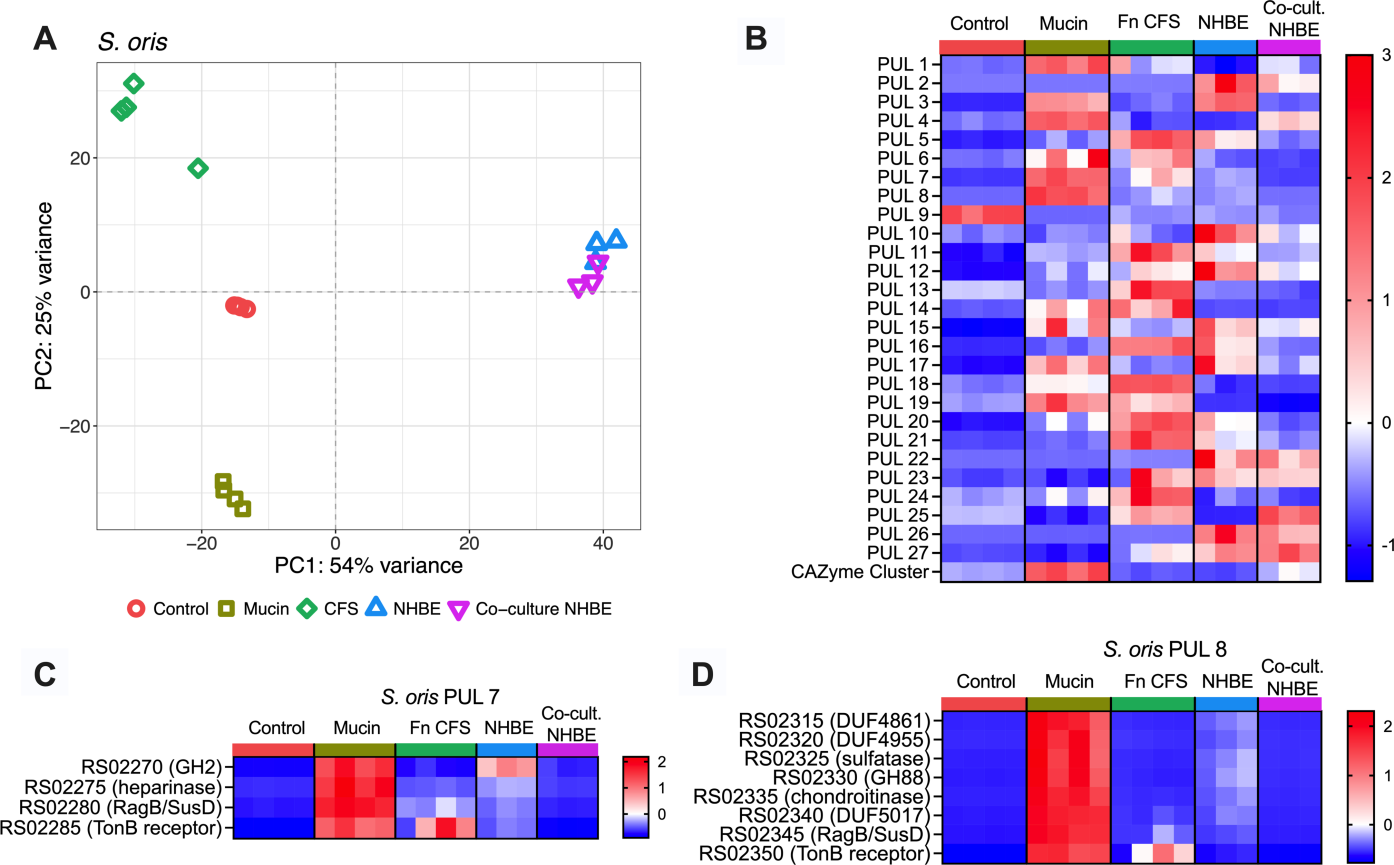
The *S. oris* transcriptome is significantly altered across media and co-culture conditions *in vitro*. (**A**) PCA of *S. oris* transcriptomes, colored by growth medium or co-culture status on NHBE, across growth conditions, lot depicting the transcriptome of individual *S. oris* samples, which are colored by growth medium or co-culture status on NHBE. Heatmaps of (**B**) average expression of every gene in a given PUL across conditions, (**C**) PUL 7, and (**D**) PUL 8 in *S. oris*. Data are scaled by row and presented as Z-scores. Statistical significance for PUL expression comparisons provided in File S4.

Both species exhibited growth phenotypes responsive to the nutritional composition of each medium (Fig. 1). Mucin supplementation modestly enhanced *F. nucleatum* growth but had a pronounced effect on *S. oris,* suggesting species-specific nutrient utilization. Growth in CFS derived from either species grown in mucin medium was comparable to or greater than growth in the control medium, indicating that neither species fully exhausted nutrients essential to the other. Interestingly, *S. oris* growth was diminished in *F. nucleatum* CFS compared to mucin medium despite *F. nucleatum* lacking known glycan-degrading capabilities. Given their potential clinical relevance and prevalence of each species in chronic airway disease, we also sought to connect broth culture data to more physiologically relevant conditions with host airway epithelia. To model how each species influences the host airway response to colonization, we employed our recently described Dual Oxic-Anoxic Co-Culture (DOAC) model, in which oxygen-dependent airway epithelial cells are supplied oxygenated blood gas basolaterally while exposing their apical surfaces to the anaerobic chamber environment (41). This advancement allows for colonization and growth of obligate anaerobes on epithelial cells, enabling study of host-pathogen-microbiota interactions. We performed RNA-seq on each bacterium in control, mucin, and CFS media, as well as dual- and triple RNA-seq on primary normal human bronchial epithelial (NHBE) cells challenged with each bacterium alone or in co-culture for 24 h. This allowed us to determine how different growth environments affect the expression of genes involved in nutrient acquisition and metabolism. Differentially expressed genes are provided in File S1 (*F. nucleatum*) and File S2 (*S. oris*) and visualized in MA plots in Fig. S2 and S6. Principal component analysis (Fig. 2A and 3A) revealed that the greatest transcriptomic separation occurred between broth cultures media and airway epithelial co-cultures, with CFS exerting the most pronounced transcriptional shift among media conditions.

### *F. nucleatum* amino acid uptake and metabolism

Since *F. nucleatum* preferentially ferments amino acids, we surveyed the expression of genes involved in amino acid uptake (Fig. 2B) and metabolism (Fig. S3) to determine which substrates might be available across each growth medium or the airway epithelial environment. Two *brnQ* homologs encoding branched-chain amino acid (BCAA) importers and a *putP* proline transporter homolog were highly expressed in control medium, moderately expressed on NHBE, and showed the lowest expression in mucin medium, *S. oris* CFS (So CFS), and NHBE co-cultures. In contrast, homologs of the *azlC* and *azlD* BCAA importer family and a sodium:alanine symporter were upregulated in mucin medium and So CFS. Interestingly, the symporter exhibited low expression on NHBE alone but was elevated when *S. oris* was co-cultured on NHBE.

Several sodium-dependent symporters and transporters were specifically induced in So CFS, while multiple ABC-type transporters and permeases were selectively upregulated in NHBE co-culture, irrespective of *S. oris* presence. A sodium:proton antiporter and GntP-family permease were strongly induced in NHBE co-culture, but only in the presence of *S. oris,* suggesting interaction-dependent metabolic shifts. Genes involved in amino acid catabolism and short-chain fatty acid production were generally upregulated in NHBE co-culture with or without *S. oris*. Among these genes were those encoding butyrate production, which have recently been linked to reactive oxygen/nitrogen resistance in *F. nucleatum* (42). Overall, while these transcriptional patterns suggest condition-specific substrate utilization, further experimental validation will be required to define precise substrate specificities.

### *F. nucleatum* carbohydrate metabolism is modulated by *S. oris*

Although *F. nucleatum* is known to catabolize glucose and fructose, other carbohydrate metabolic pathways were differentially expressed in So CFS and during co-culture with *S. oris* on NHBE cells. Because So CFS was derived from mucin medium-grown cultures, glycan degradation by *S. oris* could liberate component sugars that *F. nucleatum* subsequently metabolizes. For example, the *nan* operon (encoding a sialic acid catabolism pathway) was strongly induced in both So CFS and in NHBE co-culture conditions (Fig. 2C). Since *F. nucleatum* lacks a sialidase, its access to sialic acid is likely dependent on exogenous glycosidase activity from *S. oris*. The absence of *nan* induction in mucin medium supports this model.

*F. nucleatum* also encodes genes annotated for glycogen production and degradation though their function has not been experimentally confirmed. These genes were minimally expressed in control and mucin media but showed increased expression in So CFS and during NHBE co-culture, particularly when co-colonized with *S. oris* (Fig. 2D). This pattern suggests that glycogen-related pathways may participate in the utilization of glycan fragments generated by *S. oris*. Similarly, *F. nucleatum* encodes two putative α-1,4-polygalactosaminidases, enzymes that in some *Pseudomonas, Streptomyces,* and *Aspergillus* species degrade galactosaminoglycans such as chondroitin sulfate and dermatan sulfate (43–46). Alhough their expression was not strongly affected by the tested growth conditions, whether *F. nucleatum* homologs also degrade galactosamioglycans remains to be determined*.* More broadly, numerous genes predicted to encode carbohydrate active enzymes (CAZymes) exhibited condition-dependent expression (Fig. S4). However, most of these are not involved in host glycan degradation, as they include functions related to cell wall homeostasis (*mrcB* homolog C7Y58_RS05695) and LPS assembly or modification (*lpxCB* genes).

### Expression of *F. nucleatum* autotransporters and adhesins is modulated by nutrient and host conditions

Virulence in *F. nucleatum* is mediated by several Type V autotransporters that facilitate attachment to and invasion of host cells, and modulation of immune signaling (47–51). Therefore, we evaluated the expression of all annotated autotransporter genes across each broth and host-associated condition (Fig. S5). Expression clustered into three groups: (i) genes whose expression is highest in the control medium, (ii) those with elevated expression in mucin medium or So CFS (e.g., *radD*), and (iii) genes with maximal expression on NHBE in both mono- and co-culture. The latter group included several well-characterized *F. nucleatum* virulence factor genes, such as *fadA*, *cbpF*, fusolisin, and *fap2*. An interesting exception to this grouping was *fplA*, which was highly expressed in control, mucin medium, and on NHBE, but was repressed in So CFS and in co-culture with *S. oris* on NHBE. These data are consistent with the increasing appreciation of the connections between bacterial metabolism and regulation of virulence factors and suggest that co-colonizing *S. oris* could modulate *fplA*-mediated contributions to *F. nucleatum* virulence (52).

### *S. oris* polysaccharide utilization loci expression is responsive to both host and *F. nucleatum* interactions

The *S. oris* genome encodes 27 PULs and 1 putative CAZyme cluster, some homologous to validated systems in the *Bacteroides* genus (53, 54). Such systems are used by bacteria to sense and degrade polysaccharides typically found in plant fibers (e.g., xylan) or host glycans like mucins (55). We averaged the expression of each gene in every PUL to determine which PUL(s) respond to glycans present in mucin medium and on the apical surface of airway epithelial cells, as well as whether *F. nucleatum* supernatants or co-culture on epithelia modulate PUL expression(Fig. 3; File S3; Fig. S7). All but PUL9 were minimally expressed in control medium. Eleven PULs and the CAZyme cluster were induced in mucin medium, consistent with mucin glycan utilization. Unexpectedly, PUL expression patterns were significantly altered in Fn CFS, with decreased expression of several mucin-induced PULs and induction of multiple mucin-insensitive PULs. A third group of PULs was induced during NHBE co-culture, including PULs 3, 5, 10, 12, and 15–17, many of which were suppressed in co-culture with *F. nucleatum*. In contrast, PULs 4 and 25, minimally expressed in NHBE monoculture, were induced by *F. nucleatum*. This variable expression of PULs was anticipated between growth in mucin medium and control media and on NHBE, but modulation of PUL expression in Fn CFS and co-culture on NHBE was unexpected. These data suggest that *F. nucleatum* may influence glycan foraging by *S. oris,* supporting a dynamic bidirectional interaction.

### *S. oris* encodes a putative Type VI secretion system

Like many members of the Bacteroidota, *S. oris* encodes a predicted contact-dependent multi-protein Type VI secretion system (T6SS) that delivers toxins to adjacent bacteria in intensely competitive conditions (56). Given that *S. oris* typically resides in polymicrobial settings and, therefore, could plausibly benefit from such a system, we scanned the *S. oris* NCTC 13071 genome for T6SS genes using SecReT6 (File S4) (57). Three genomic loci were identified, including one containing 13 clear homologs of known T6SS genes and 2 smaller clusters each containing 4 homologs. Expression profiles (Fig. S8) revealed moderate expression in control medium and mucin media, with higher expression of select genes in Fn CFS. The *hcp* homolog was only expressed in control medium, while the *tssC* homolog was only expressed on NHBE, irrespective of the presence of *F. nucleatum*. A *tssI* homolog (locus tag EL210_RS03325) exhibited low-to-moderate expression across broth cultures and minimal expression in monoculture on NHBE but was highly induced in NHBE co-culture with *F. nucleatum,* suggesting potential functional activation in response to interspecies contact. Given the lack of coordinated expression, it is unclear whether they comprise a functional T6SS and will require further experimental validation.

### *F. nucleatum* drives airway epithelial inflammation, modulated by *S. oris* co-colonization

**Fig 4:**
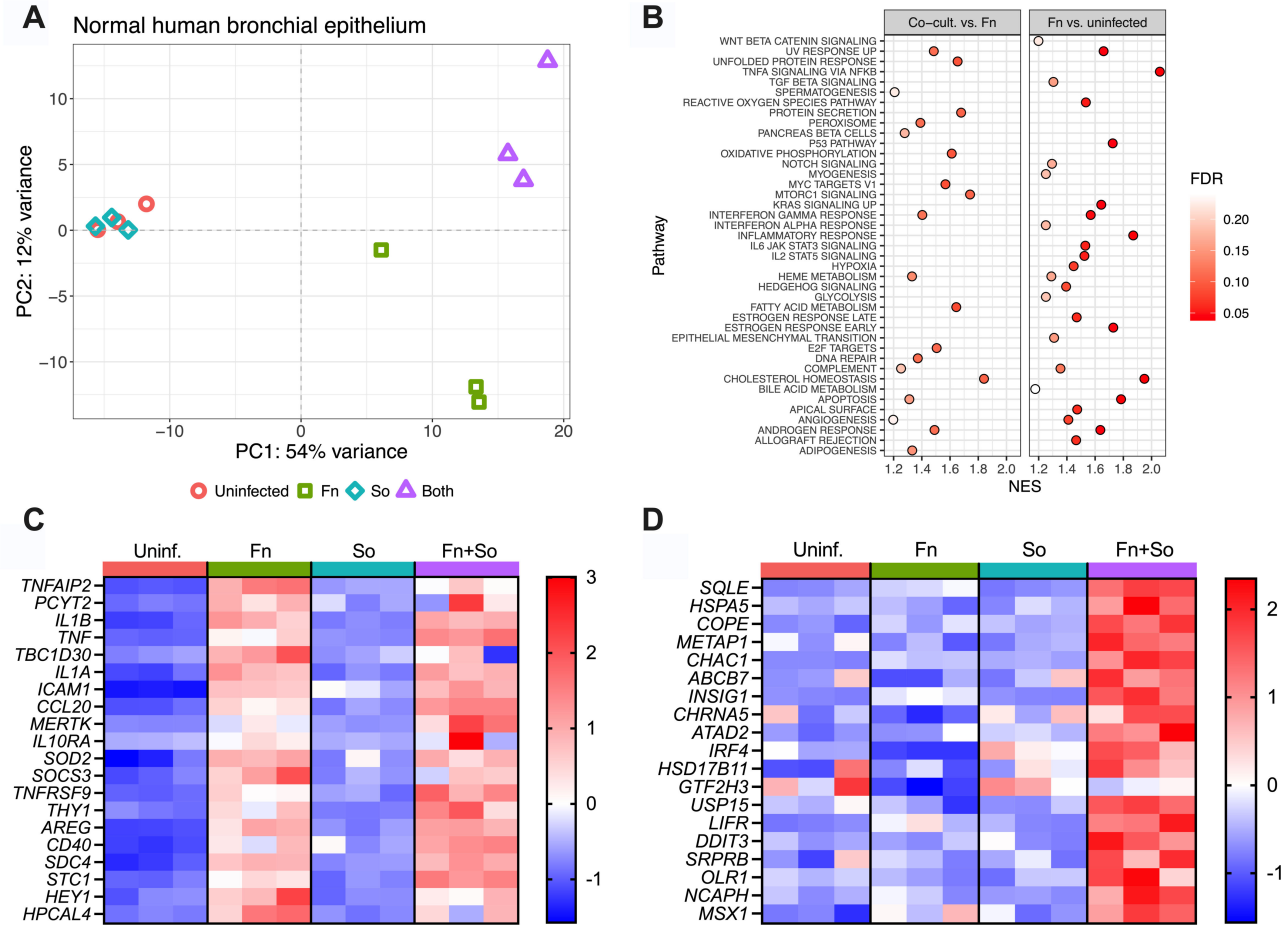
Bacterial colonization alters the NHBE transcriptome. (**A**) PCA plot of host transcriptomes following mono- and co-colonization with *F. nucleatum* and *S. oris.* (**B**) Gene set enrichment analysis (GSEA) in samples from NHBE colonized with both *F. nucleatum* and *S. oris* compared to mono-colonization with *F. nucleatum* (left) and NHBE mono-colonized with *F. nucleatum* compared to uninfected cells. Normalized enrichment scores (NES) are shown on the *x*-axis, and the color of each point indicates false discovery rate (FDR). High NES with low FDR indicates a pathway enriched in a given comparison. Pathways enriched in both comparisons were removed from the Co-cult versus Fn plot to highlight pathways unique to this comparison, and only those pathways with an FDR < 0.25 are shown. Expression heatmaps of transcripts enriched in (**C**) cells mono-colonized with *F. nucleatum* or (**D**) cells dual-colonized with *F. nucleatum* and *S. oris*. Data in panels C and D are scaled by row and presented as Z-scores.

We next profiled the transcriptional response of NHBE cells following 24 h colonization with *F. nucleatum* or *S. oris,* alone or in co-culture, relative to uninfected controls (Fig. 4A; Fig. S9, File S5). As expected, several mucin genes were expressed across all conditions, consistent with the presence of an epithelial mucus layer (Fig. S10). PCA analysis revealed that *S. oris* alone had little effect on the host transcriptome, whereas *F. nucleatum* induced a distinct and divergent host response. Co-colonization clustered separately from *F. nucleatum* monocolonization, suggesting *S. oris* modulates epithelial responses either directly or indirectly via interactions that modulate *F. nucleatum* virulence.

Gene set enrichment analysis (GSEA) (Fig. 4B; Files S6 and S7) identified enrichment of inflammation- and cancer-associated pathways in *F. nucleatum*-colonized epithelia, including TNF-α, IL-6/JAK/STAT3 signaling, IL-2/STAT5 signaling, and Wnt/β-catenin signaling. Anti-inflammatory pathways were also suppressed, including downregulation of *HAS2*, which encodes hyaluronan synthase. Because hyaluronan is protective against airway inflammation and hyperresponsiveness, its suppression by *F. nucleatum* may exacerbate these processes in diseased airways (58).

Co-colonization with *S. oris* altered the NHBE transcriptome relative to monocolonization with either species, underscoring the influence of microbial interactions on host inflammatory signaling. For example, the most highly induced gene in co-colonized NHBEs relative to *F. nucleatum* alone was *PLEKHF2,* whose product is involved in micropinocytosis and TNF-driven apoptosis (59). *EDEM3,* associated with the unfolded protein response and endoplasmic reticulum stress (60), was also upregulated during co-colonization. Interestingly, *EDEM3* has been reported to translocate to the HeLa cell surface in response to the pore-forming listeriolysin O toxin from *Listeria monocytogenes* (61), suggesting possible parallels in epithelial stress responses.

Co-culture of both species on NHBE also influenced the expression of genes that were uniquely enriched relative to *F. nucleatum* mono-colonization (Fig. 4C and D). Some inflammatory markers, such as *IL1-*β*,* were induced by *F. nucleatum* alone and did not change in co-culture, while others, including *SQLE* (encoding squalene epoxidase), were upregulated only under co-colonization conditions. Together, these data reveal that (i) *F. nucleatum* elicits a strong pro-inflammatory transcriptional program in airway epithelial cells and (ii) co-colonization with *S. oris* further reshapes this response, highlighting complex, interspecies interactions that modulate host inflammation in ways not apparent during monocolonization.

## DISCUSSION

The frequent co-colonization of *F. nucleatum* and *S. oris* across mucosal environments suggests potential functional interactions that influence bacterial colonization, persistence, and host responses. Using both *in vitro* and airway epithelial culture models, we show that these species engage in bidirectional interactions involving glycan degradation, metabolite exchange, and transcriptional modulation. Although some transcriptional overlaps were observed, the global gene expression profiles of both species in broth culture differed markedly from those during epithelial colonization, particularly in pathways related to metabolism and *F. nucleatum* virulence regulation. These differences likely reflect two non-mutually exclusive factors: (i) the distinct physical and nutritional environments of liquid culture vs the static, host-associated surface of NHBEs and/or (ii) variation in bacterial growth phase between conditions. Although CFUs were not measured for either species on NHBEs to directly assess growth phase, these findings underscore the importance of modeling microbial interactions under physiologically relevant conditions. Transcriptomic analyses were performed on 24 h cultures corresponding to early stationary phase for both species (Fig. 1A and D), a time point that captured active metabolism while minimizing nutrient depletion. Nonetheless, inherent growth differences between *F. nucleatum* and *S. oris* may also contribute to the observed variability in gene expression.

Despite these contextual differences, two transcriptional signals indicative of glycan-mediated interactions were consistently observed across both culture and host-associated conditions. First, the *F. nucleatum nan* operon, which encodes a sialic acid catabolism pathway, was not expressed in mucin medium but was strongly induced in *S. oris* supernatants and during co-colonization on NHBE cells. Given that *F. nucleatum* lacks sialidase activity, these data support a model in which sialic acid utilization depends on glycosidase activity from co-resident microbiota. This finding is consistent with previous work (28) that showed that *F. nucleatum* exploits sialidase-positive species to access host-derived sialic acids in dysbiotic vaginal communities. Although the *nan* operon is not conserved across all *F. nucleatum* subspecies, the broader concept of *F. nucleatum* promoting dysbiotic bacterial community structures through metabolic cross-feeding likely extends to multiple mucosal environments. Indeed, Queen et al. identified *F. nucleatum* within polymicrobial biofilms on colorectal tumors and noted enrichment of sialic acid-adjacent metabolic pathways (62). Similarly, *F. nucleatum* was shown to bind to and interact with *Clostridioides difficile* on MUC2-coated surfaces in a bioreactor model, promoting biofilm formation and broad transcriptional changes in *C. difficile*, including metabolic reprogramming (63). Furtado et al. further observed induction of a fructose/mannose PTS system when *C. difficile* was grown in the mucus layer of intestinal epithelial cells *in vitro* (64)*.* Interestingly, we also detected increased expression of a homologous system in *F. nucleatum* grown in mucin. Although our study focused on transcriptional responses, future biochemical analyses of cell-free supernatants (e.g., LC-MS) will be important to identify specific metabolites exchanged between species and to validate the cross-feeding interactions inferred here.

A second glycan-associated transcriptional signal involved modulation of *S. oris* PULs. While several PULs were robustly induced by mucin medium, their expression was suppressed in the presence of *F. nucleatum* supernatants or during co-colonization on epithelial cells. This is notable for two reasons: (i) the large number of PULs affected and (ii) the fact that *F. nucleatum* has not been reported to degrade host-associated glycans and is, therefore, not expected to appreciably alter the glycan landscape of mucin medium. Porcine gastric mucin used in this study is a crude proteolytic digest and likely contains non-mucin glycans such as glycosaminoglycans, which may explain the broad induction of PULs. For example, PULs predicted to target heparin and chondroitin (e.g., PULs 7 and 8) were induced in both mucin medium and on NHBE cells but were repressed in the presence of *F. nucleatum,* suggesting nutrient depletion or transcriptional repression. One possible mechanism is that *F. nucleatum* secretes metabolic byproducts that modulate *S. oris* gene expression. These products could include secreted polyglucose or glycogen, which could then be available to *S. oris* or scavenged from dead *F. nucleatum* cells. Notably, *F. nucleatum* glycogen metabolism genes were upregulated on NHBE, particularly during co-culture with *S. oris*, but not in mucin medium, suggesting that glycogen is unlikely to be present in *Fusobacterium* supernatants.

The airway epithelial response was strongly influenced by *F. nucleatum* and further modulated by co-colonization with *S. oris.* Consistent with prior single-cell RNA-seq studies (41), *F. nucleatum* elicited robust inflammatory signaling, including induction of *TNF-α, IL-1β,* and mitochondrial stress responses suggestive of apoptosis. In contrast, NHBE colonized with *S. oris* alone exhibited no differentially expressed genes compared to controls, consistent with its commensal nature. Nevertheless, co-colonization with *S. oris* altered the epithelial response, indicating that microbial interactions can modulate host sensing and inflammation. For example, *TNS4*, a gene linked to poor prognosis in lung adenocarcinoma (65), was significantly downregulated in NHBEs co-colonized with *S. oris* and *F. nucleatum* relative to *F. nucleatum* alone. Conversely, co-culture induced expression of several genes not responsive to either bacterium individually (Fig. 4D). Because *S. oris* alone had minimal effect, we hypothesize that co-colonization alters *F. nucleatum* behavior in ways that modulate host responses. However, we cannot rule out *F. nucleatum*-driven changes in *S. oris* behavior that directly influence the airway epithelial response. Regardless, these data collectively highlight the need to consider microbial interactions when modeling host-pathogen dynamics and support integrating microbiome-derived hypotheses with mechanistic approaches to uncover host-pathogen-microbiome interactions.

In summary, our findings demonstrate that interactions between nutrient availability, the host environment, and co-colonizing bacteria shape the behavior of *F. nucleatum, S. oris,* and airway epithelial cells. Limitations should be acknowledged. While transcriptional and translational outputs are generally correlated, differences in mRNA and protein stability may result in temporal or spatial discrepancies in bacterial and host behavior that are not captured here. Likewise, site-directed mutagenesis is required to confirm several bacterial interactions suggested by our data, but genetic tools for *F. nucleatum* are only recently emerging and remain unavailable for *S. oris*. Finally, we evaluated only one strain of each species, which constrains our ability to assess strain- and subspecies-level genetic (and thus phenotypic) diversity. Despite these limitations, our results provide a framework for exploring glycan-driven cross-feeding and its impact on pathobiont behavior and host responses in mucosal environments.

## Data Availability

All sequencing files are available at the NCBI Sequence Read Archive (SRA) under accession number PRJNA1282640.

## SUPPLEMENTAL MATERIAL

File S1 msystems.00922-25-s0001.xlsx All differentially expressed genes in *F. nucleatum* across the growth conditions in this study.

File S2 msystems.00922-25-s0002.xlsx All differentially expressed genes in *S. oris* across the growth conditions in this study.

File S3 msystems.00922-25-s0003.xlsx ANOVA results comparing averaged PUL in *S. oris* across all growth conditions in this study.

File S4 msystems.00922-25-s0004.xlsx *S. oris* genes predicted by SecReT6 to be homologs of type 6 secretion system genes.

File S5 msystems.00922-25-s0005.xlsx GSEA of NHBE incubated with *F. nucleatum* for 24 hours compared to uninfected NHBE.

File S6 msystems.00922-25-s0006.xlsx GSEA of NHBE incubated with *F. nucleatum* for 24 hours compared to uninfected NHBE.

File S7 msystems.00922-25-s0007.xlsx GSEA of NHBE incubated with *F. nucleatum* and *S. oris* for 24 hours compared to NHBE incubated with *F. nucleatum* alone.

Supplemental Figures msystems.00922-25-s0008.pdf Figures S1 to S10.

ASM does not own the copyrights to Supplemental Material that may be linked to, or accessed through, an article. The authors have granted ASM a non-exclusive, world-wide license to publish the Supplemental Material files. Please contact the corresponding author directly for reuse.
