# Supplementary material for: Cross-feeding interactions between Fusobacterium nucleatum and the glycan forager Segatella oris
Source: mSystems. 2026 Jan 21;11(2):e00922-25. doi: 10.1128/msystems.00922-25 (PMC12911393; doi:10.1128/msystems.00922-25)
Supplement: Supplemental Figures — Figures S1 to S10. [file msystems.00922-25-s0008.pdf]

Cross-feeding interactions between *Fusobacterium nucleatum* and the glycan forager *Segatella oris*

Supplemental Data

J.R. Fletcher et al.

Document S1. Figures S1-S10

File S1. *F. nucleatum* differentially expressed genes

File S2. *S. oris* differentially expressed genes

File S3. *S. oris* PUL ANOVA p-values

File S4. *S. oris* SecReT6 predicted T6SS genes

File S5. NHBE differentially expressed genes

File S6. NHBE + Fn vs. NHBE untreated differentially expressed genes

File S7. NHBE + Fn + So vs. NHBE untreated differentially expressed genes

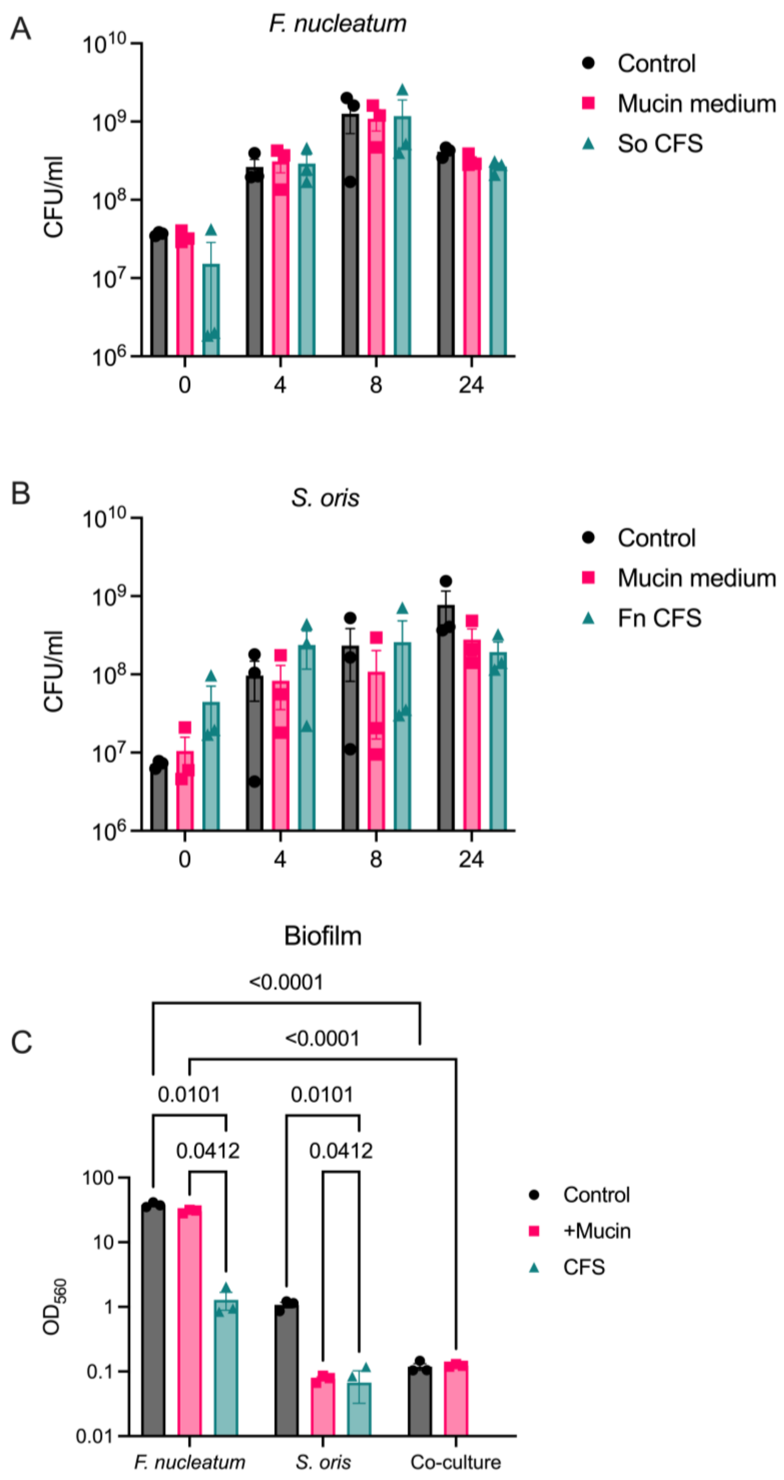

Fig. S1. ***F. nucleatum* and *S. oris* CFUs and co-culture biofilms *in vitro*.** A and B) CFUs of (A) *F. nucleatum* and (B) *S. oris* grown in different media over time. (C) *F. nucleatum* and *S. oris* were grown together in control and mucin medium and biofilms were stained with crystal violet and quantified at OD560. *F. nucleatum* and *S. oris* monoculture biofilms from Fig. 1 are included in this graph for comparison.

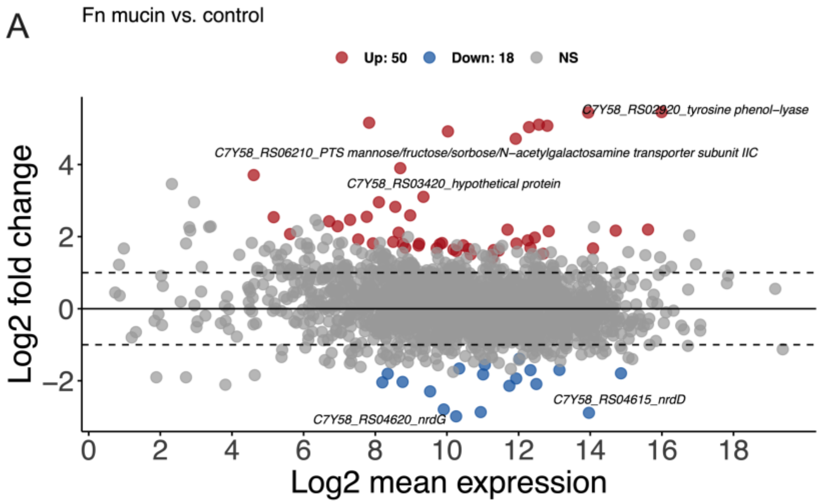

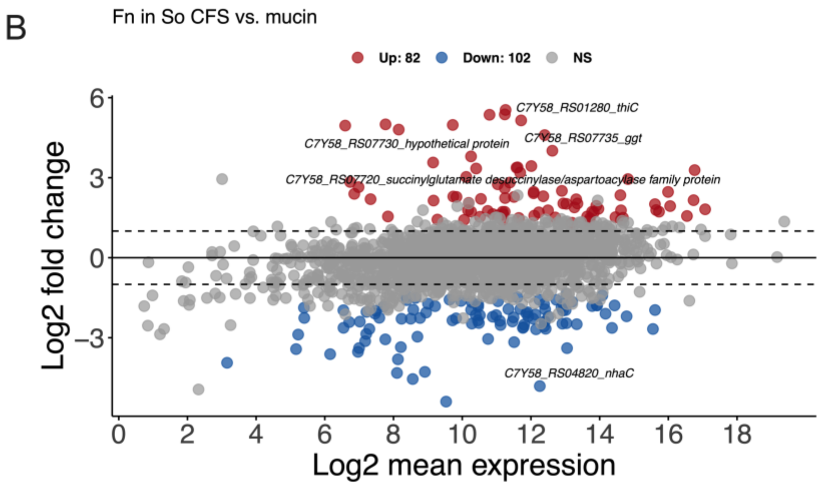

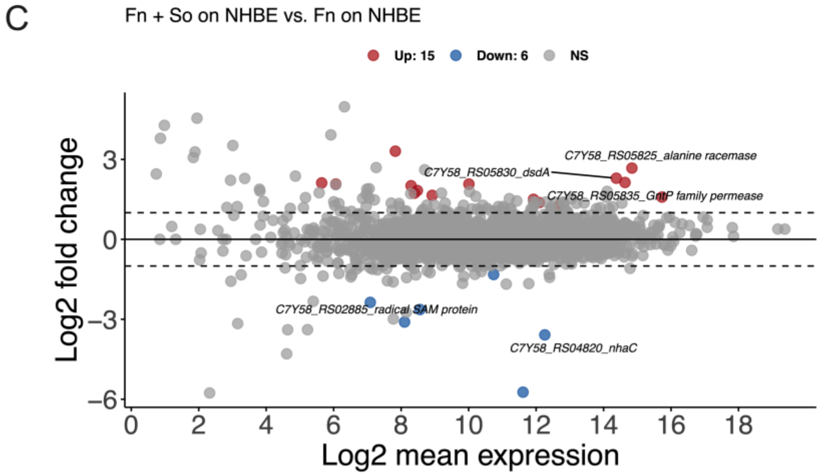

Fig. S2. MA plots of *F. nucleatum* RNA seq comparisons in A) mucin medium relative to the control medium, B) *S. oris* mucin medium 48-hour cell-free supernatants (CFS) relative to mucin medium, C) *F. nucleatum* in 24-hour co-culture with *S. oris* on primary normal human bronchial epithelia (NHBE) compared to *F. nucleatum* 24 hour monoculture on NHBE.

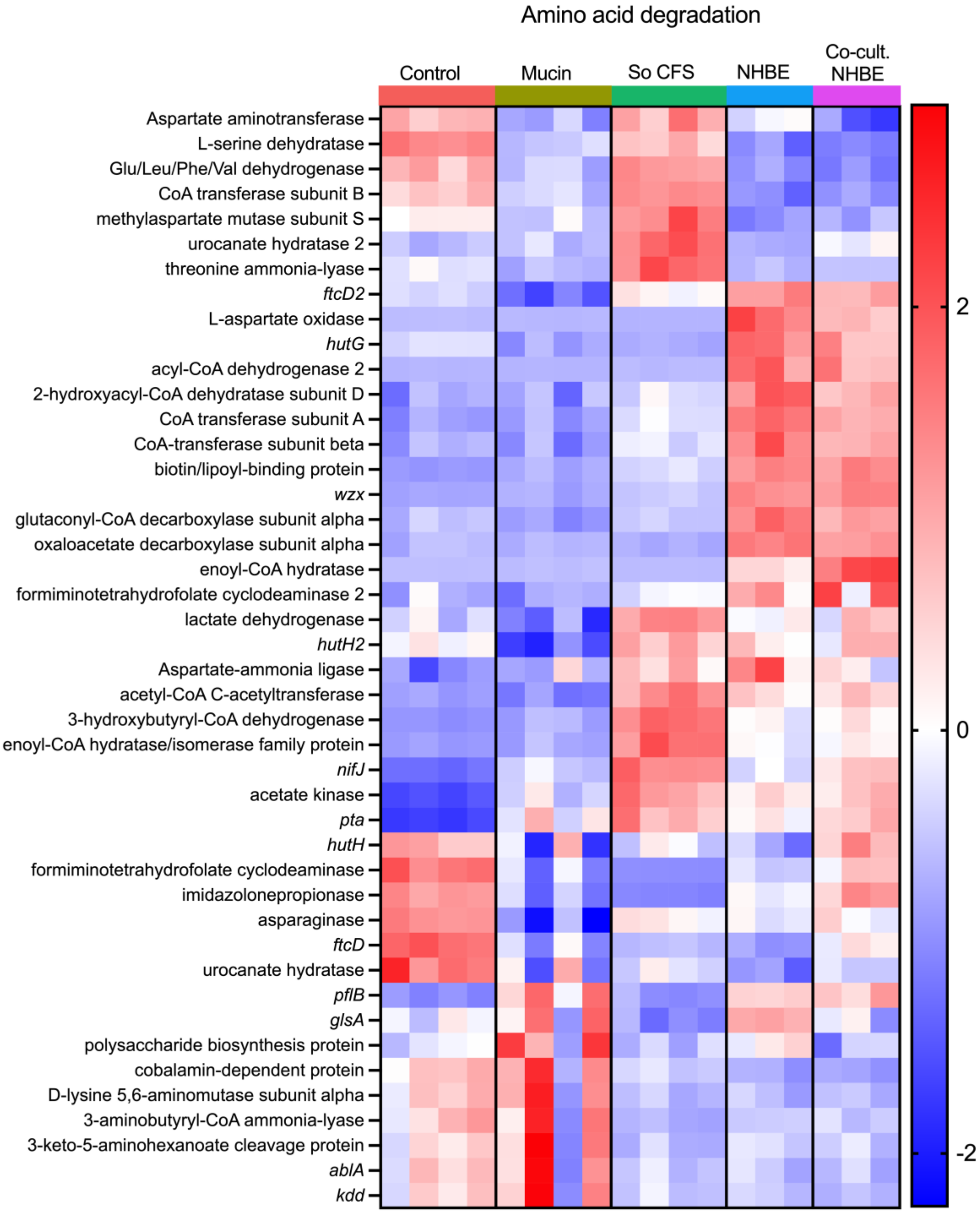

Fig. S3. *F. nucleatum* amino acid degradation gene expression across various media and on NHBE with and without co-culture with *S. oris*. Data are scaled by row and presented as Z-scores.

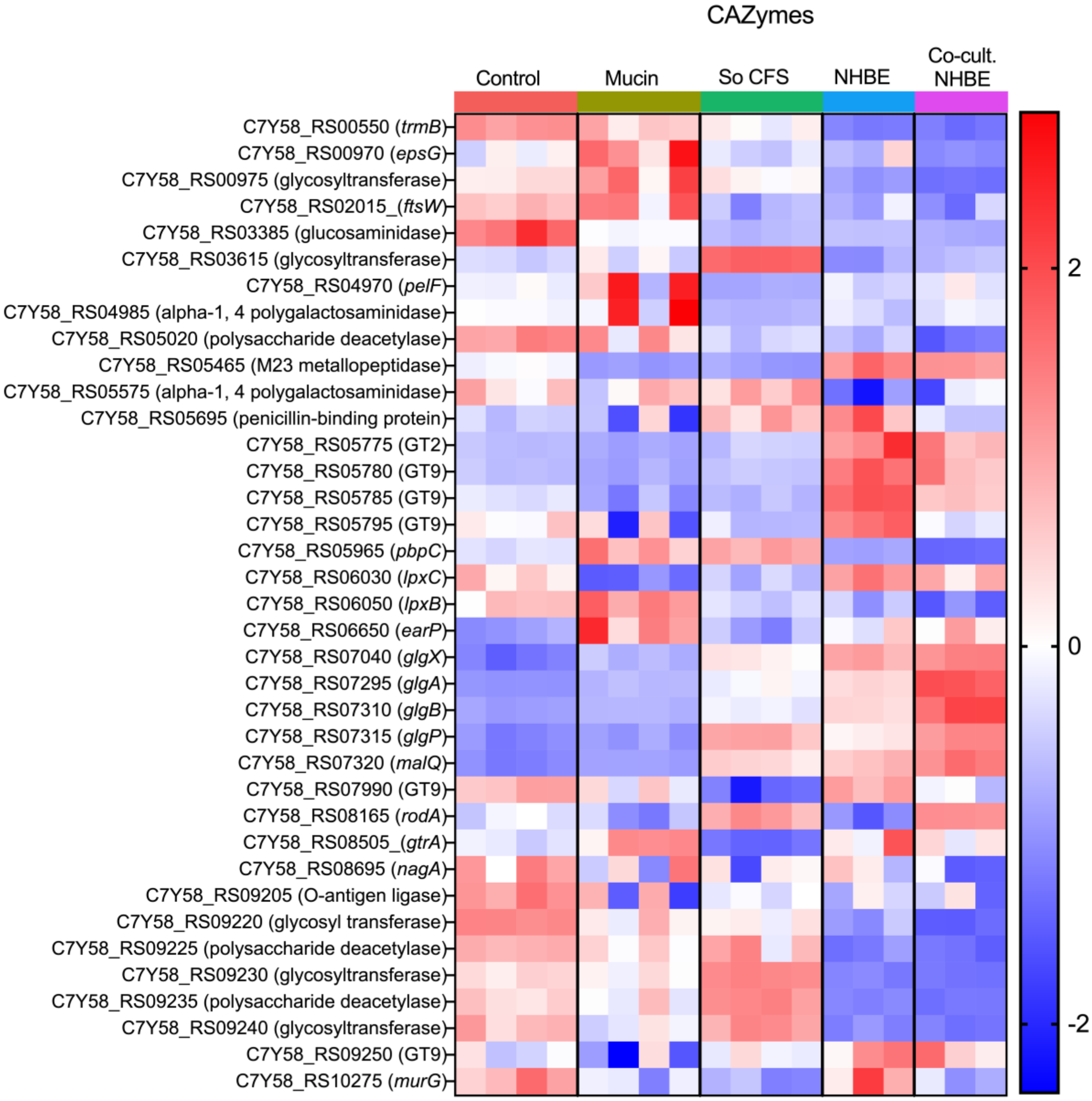

Fig. S4. Expression of *F. nucleatum* carbohydrate active enzymes (CAZymes) across various media and host conditions. Data are scaled by row and presented as Z-scores.

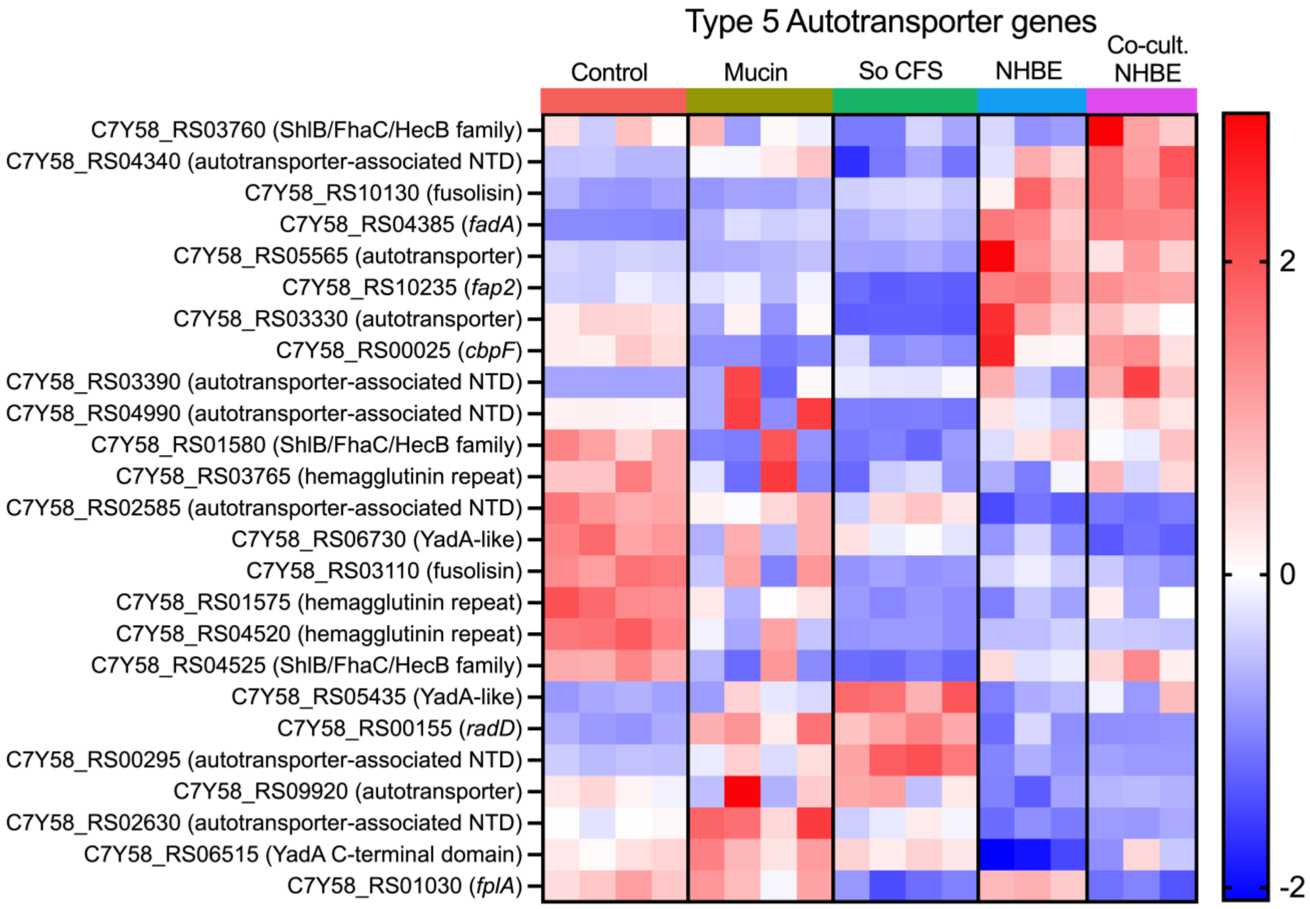

Fig. S5 - Expression of known and putative *F. nucleatum* autotransporters, adhesins, and virulence factors. Data are scaled by row and presented as Z-scores.

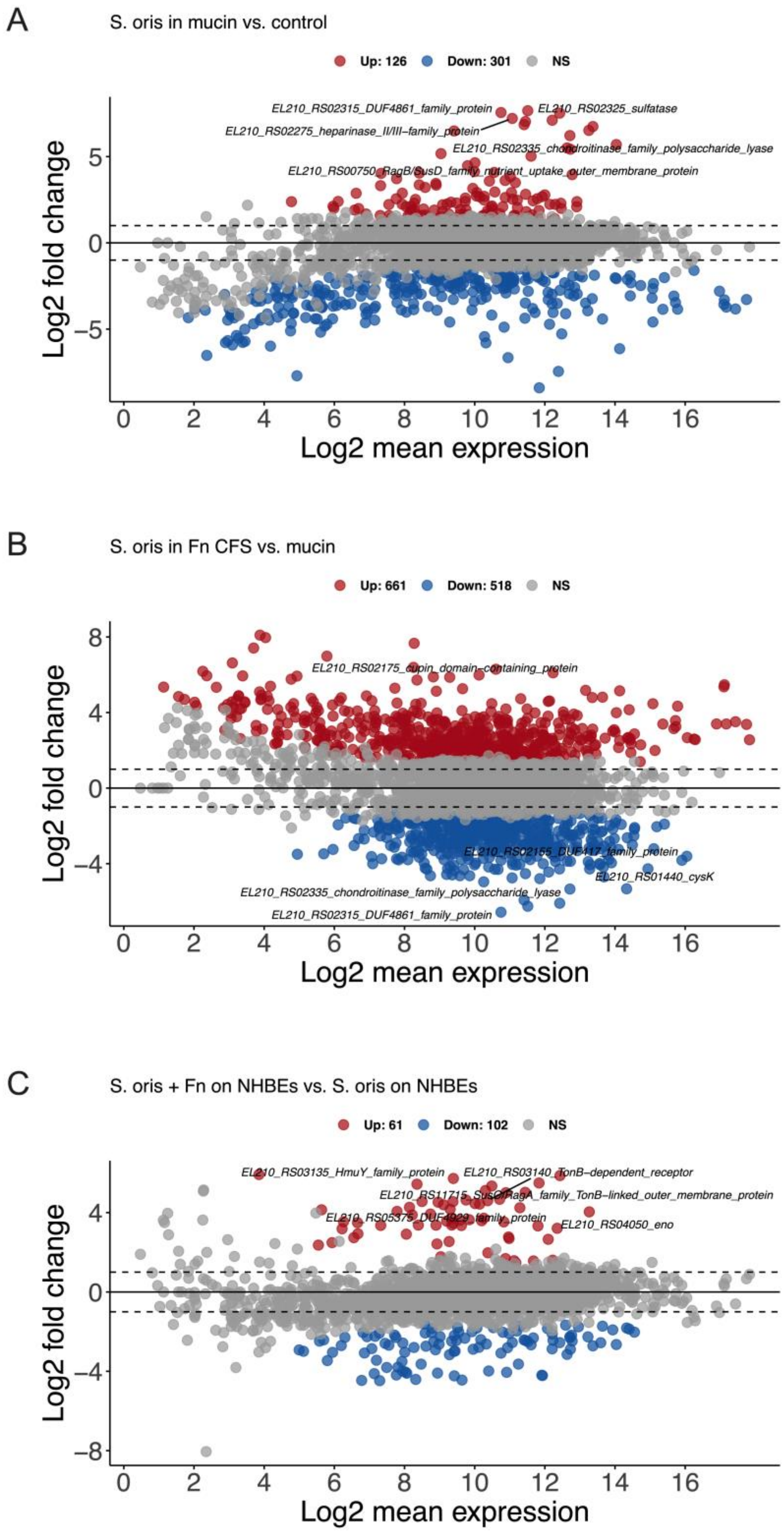

Fig. S6. MA plots of *S. oris* RNA seq comparisons in A) mucin medium relative to the control medium, B) *F. nucleatum* 48-hour mucin medium cell-free supernatants (CFS) relative to mucin medium, C) *S. oris* in 24-hour co-culture with *F. nucleatum* on primary human normal bronchial epithelia (NHBE) relative to *S. oris* monoculture on NHBE.

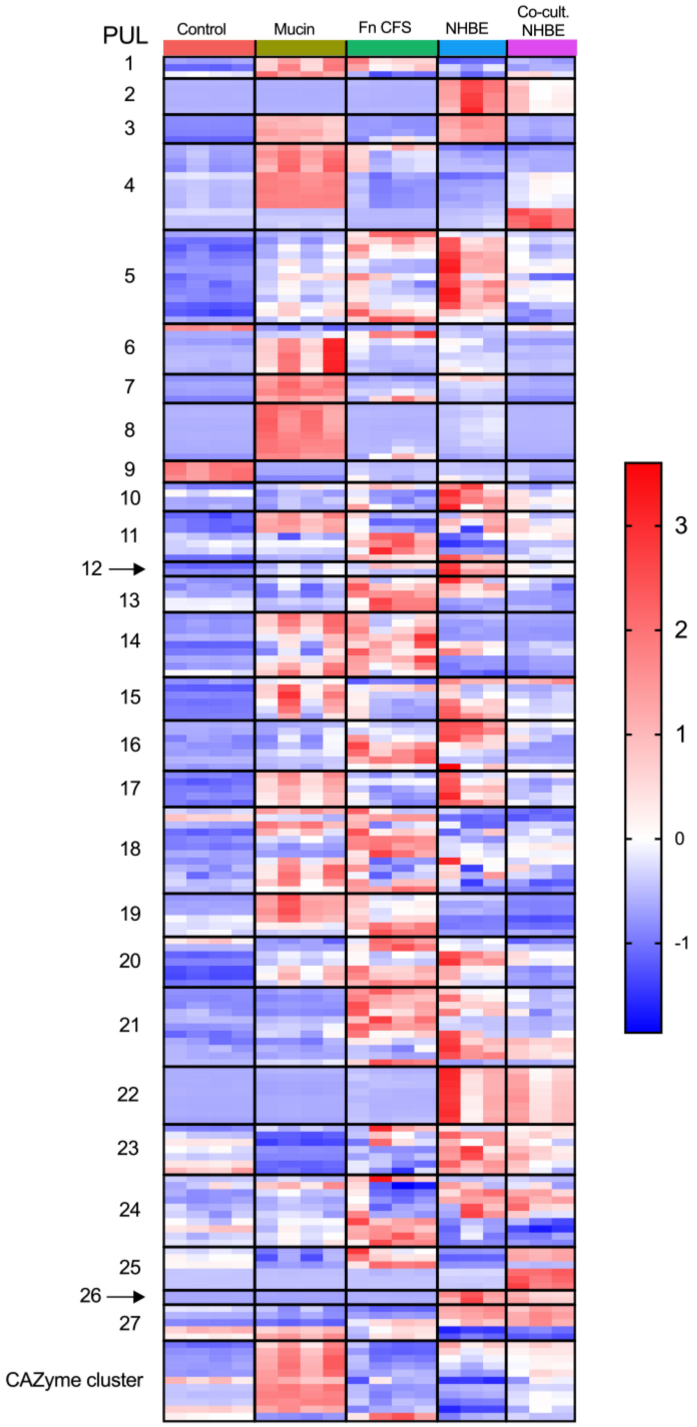

Fig. S7. *S. oris* polysaccharide utilization loci (PUL) expression across all conditions. Data are presented as Z-scores scaled by row.

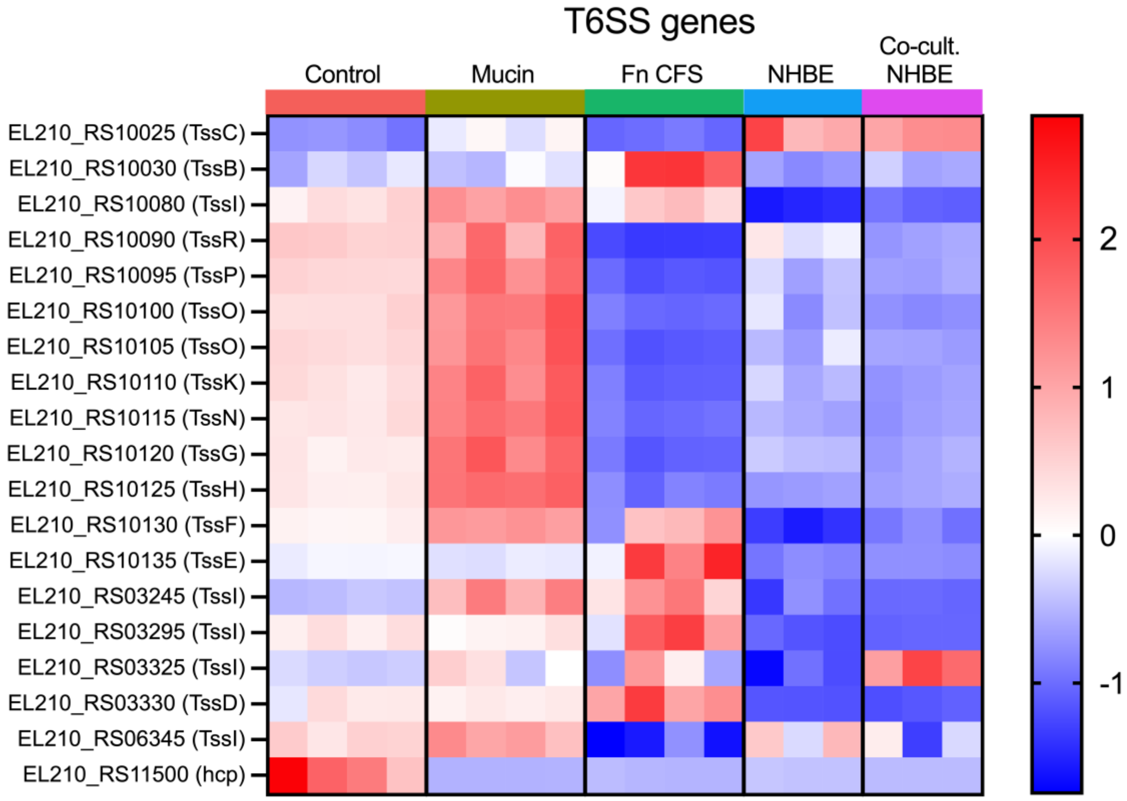

Fig. S8 – Expression of *S. oris* putative Type VI Secretion System genes across all conditions. Data are presented as Z-scores scaled by row.

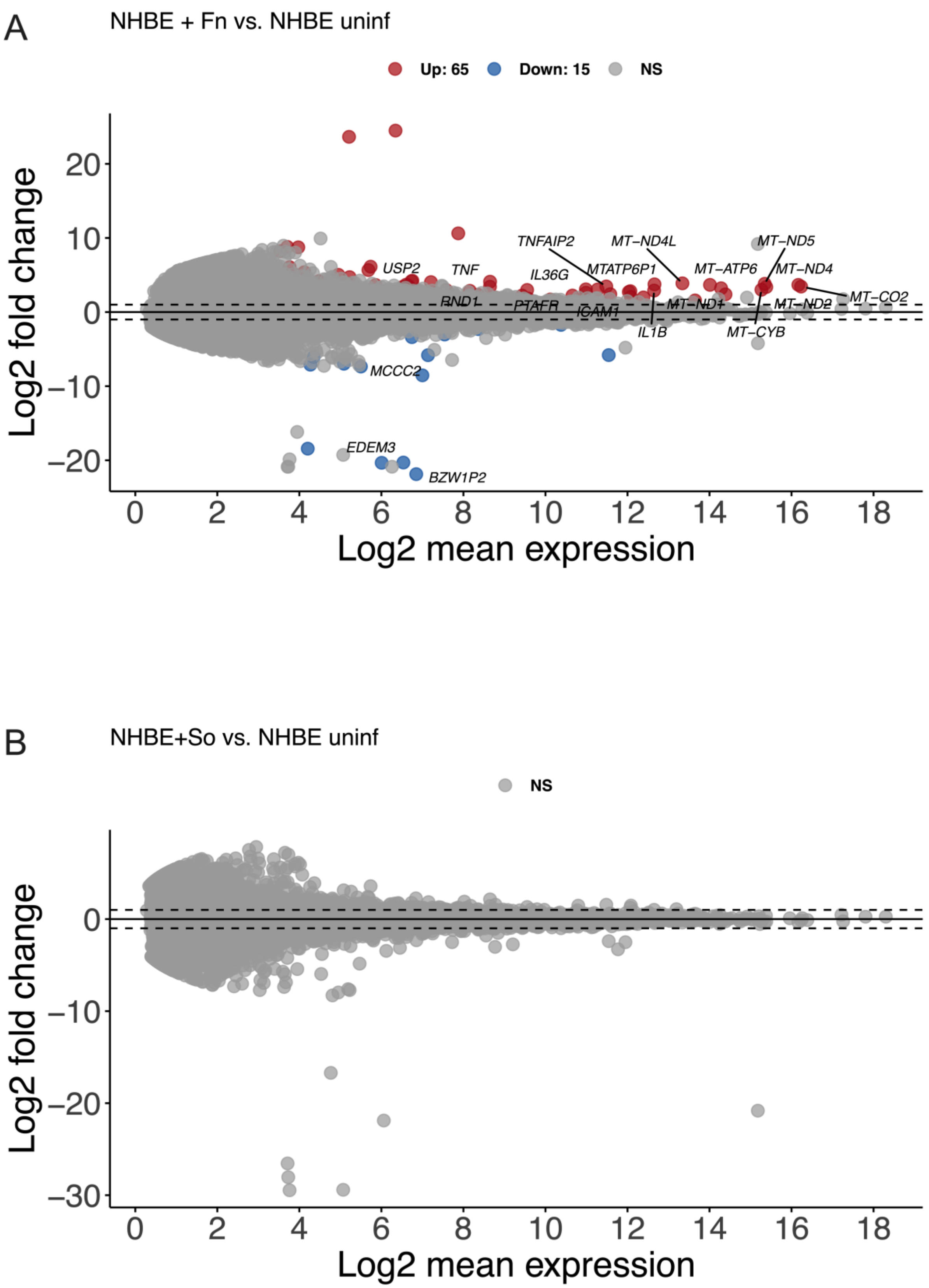

Fig. S9 – MA plots of RNA seq of primary normal human bronchial epithelia (NHBE) colonized for 24 hours with A) *F. nucleatum* relative to uninfected control NHBE, B) *S. oris* relative to uninfected controls. C,D on the next page.

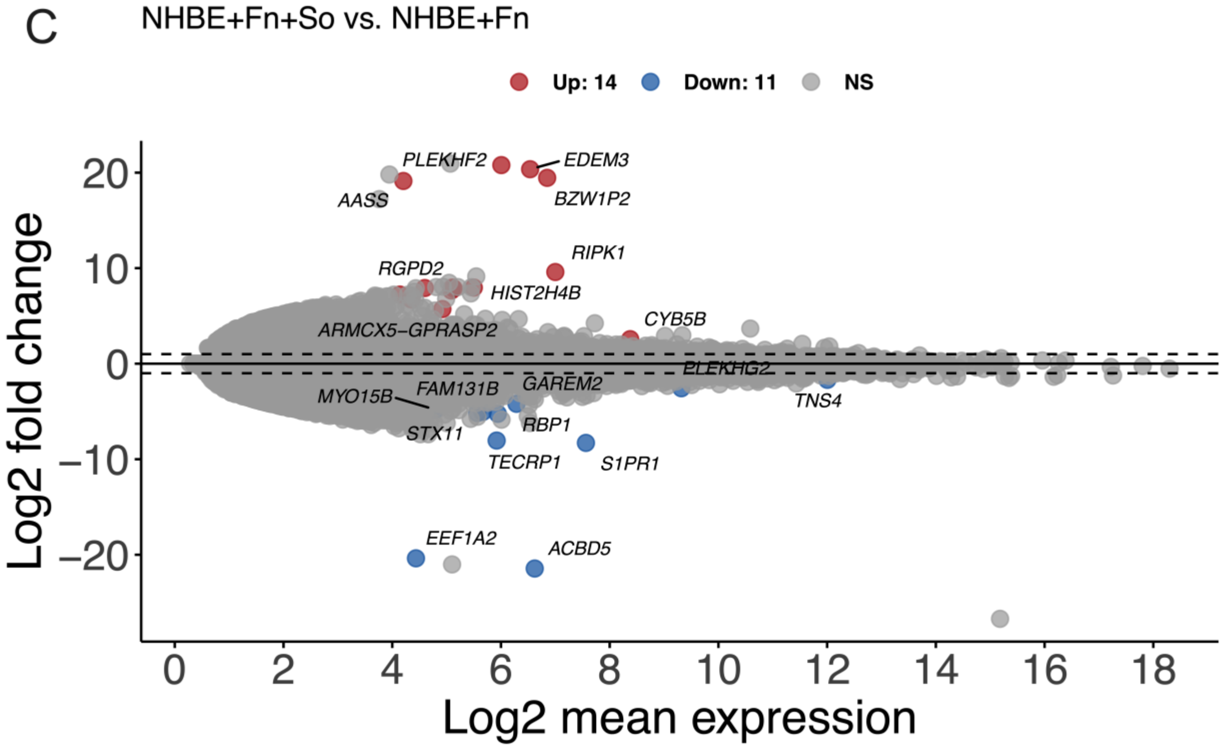

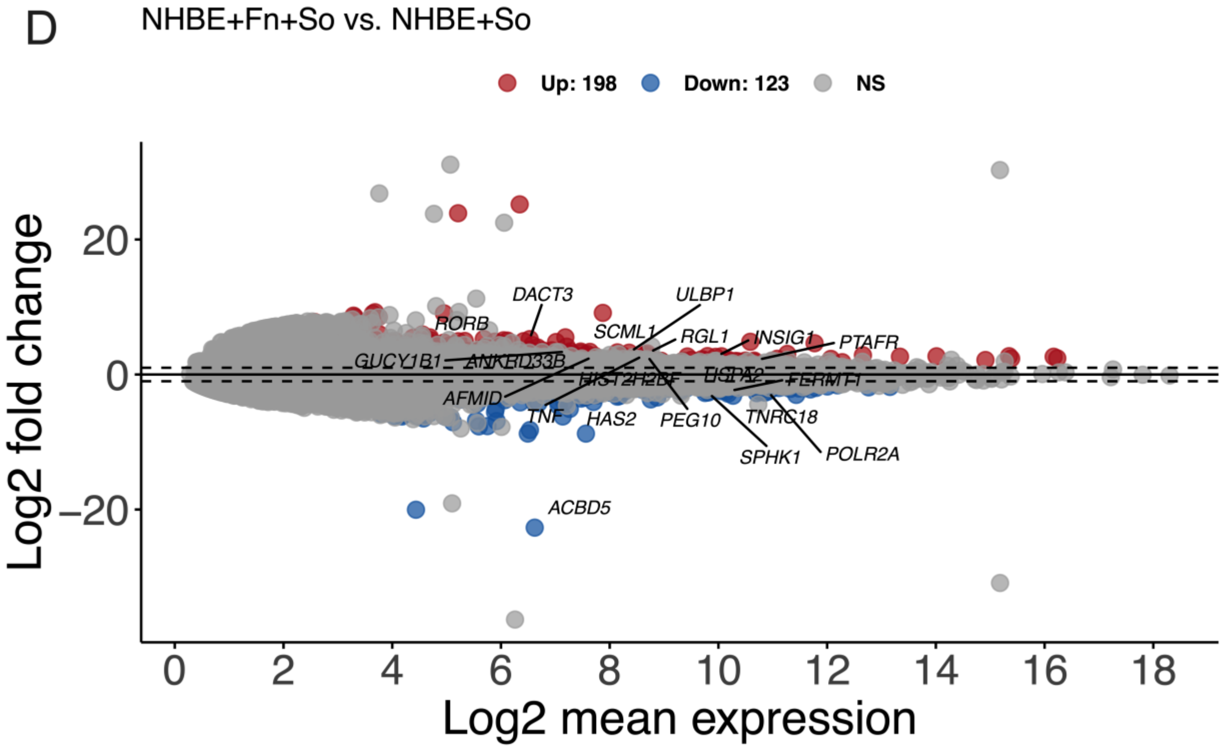

Fig. S9 continued. C) *F. nucleatum* and *S. oris* relative to monocolonization with *F. nucleatum*, and D) *F. nucleatum* and *S. oris* relative to monocolonization with *S. oris*.

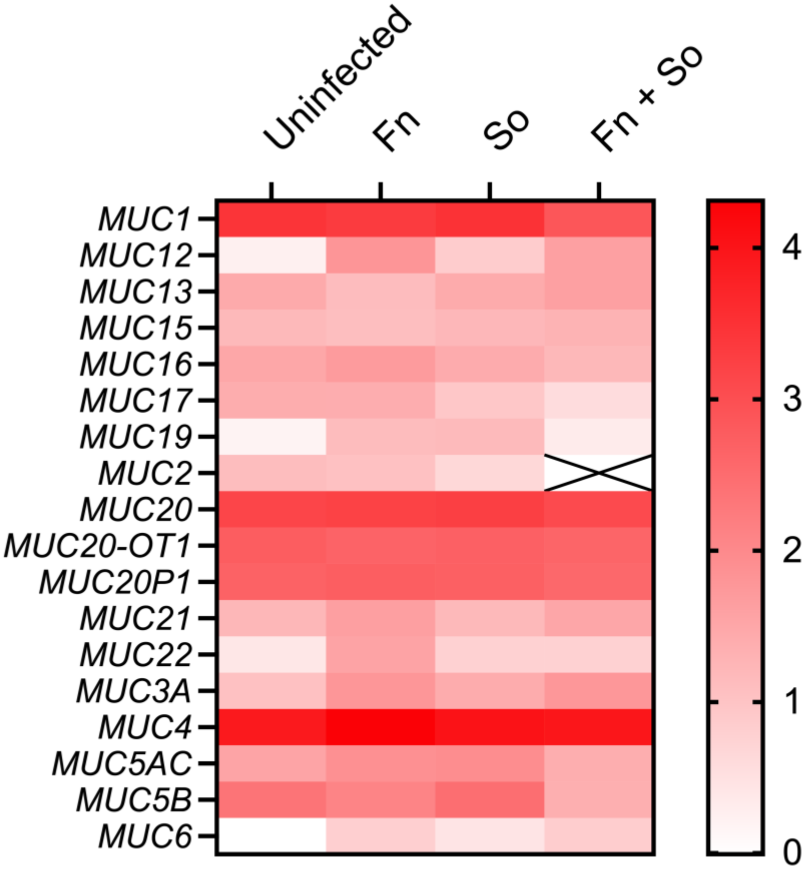

Fig. S10 – Heatmap of log10 transformed mucin gene normalized read counts in NHBE across all conditions. Darker red indicates higher expression.
